# Supplementary material for: Identification of Male-Specific Molecular Markers by Recombination of RhoGEF10 Gene in Spotted Knifejaw (Oplegnathus punctatus)
Source: Genes (Basel). 2022 Jul 15;13(7):1262. doi: 10.3390/genes13071262 (PMC9317648; doi:10.3390/genes13071262)
Supplement: Supplementary file 1 [file genes-13-01262-s001.zip › Supplementary File S1.pdf]

Base sequence of female *Oplegnathus punctatus* (326 bp)

1 CAACTACCAC TCTGAATGCT GCTTAATTTA TTGTTGGAGG CCAGTGTTAA GAGATTTAAG

61 GACTGGTTCT CTTTACCTTC TGCTCTCTTA AGTAGGTGCA AATGTGAACT TTTATGTGAT

121 TTAGAAGGTG TTTTCACACA ATTTAATTGC TGTATGTTGA AACTAATGAG TTGACTTGAT

181 ATTTGTTCTT TCAGTTCTGT CATGAAAAGT CAACTCTTGG GTTGATCCGT ACAAGACGGA

241 TAAATCAAGG CTCGAAAATA AAACATTTGT TTTAAAATAT GAAAATACAA GCTTGAAACA

301 ATGGGCCTAA AGTGTGGTGG TTGAAC

Base sequence of male *Oplegnathus punctatus* (879 bp)

1 CAACTACCAC TCTGAATGCT GCTTAATTTA TTGTTGTAGG CCAATGTTAA GAGATTTAGG

61 GTGCTTTCAC ACCTGAGCTG TTTGGTCCGT TTTAATTGAA CCCTGGTGCA TTCGTTGGAT

121 AGTCCGGTTT GTTTGGGGTG GTGTGAAAGC TCATTCGAAA TCCAAACAAC TTTCAAATCC

181 ATAGCTTATT ATGATCAAAT CTCGCCCGTC TTCTGTCTAA CAACGACAAC ACATCATAAT

241 CCCTCTCTCT CATCAGGGCC TGCCTTCTCC TTCTCACTTG CTGTCTTGTC AGCTGCTCAT

301 ATTGTTTGCC TTCTTGTAAA GCATTTAATT GATGGAAACA CCAAAGCAAA ACCACAAAAA

361 CTCAAGACCA CATAAATTTG GTGTTGTTCC ATTATTCCTG AGATCAGGAA GTGTCGGCGA

421 GTTTCACTCG GATATTTTGT CCAATTGACG AGCAACCGTT TTGACCGCGT GACATTTGTT

481 TACAATATTT GGTCTGCTTG ACAAAGTTAC AGTGTGAAAG CGAACCGAAC CAAATGAAAA

541 ATGCAACAAT GTTGCAACTT CACCCCGAAT CGCTCCGAGA CCACCGAACT ACAGATGTGA

601 AAGCGCCCTT AAGGGCAGGT TCTCTTTACC TTCTGCTCTC TTAAGTATGT GCAAATGTGA

661 ACTTTTATGT GATTTAGAAG GTGTTTTCAC ACAATTTAAT TGCTGAATGT TGAAACTAAT

721 GAGTTGACTT GATCTTTGCT TTTTCAGTTC TGTCATGAAA TGTCAACTCT TGGGTTGATC

781 CGTACAAGAC GGTTAAATCA CGGCTCAAAA ATAAAACATT CGTTTTAAAA TATGAAAATA

841 CAAGCTTGAA ACAATGGGCC TAAAGTGTGG TGGTTGAAC
