# Supplementary material for: Identification of Male-Specific Molecular Markers by Recombination of RhoGEF10 Gene in Spotted Knifejaw (Oplegnathus punctatus)
Source: Genes (Basel). 2022 Jul 15;13(7):1262. doi: 10.3390/genes13071262 (PMC9317648; doi:10.3390/genes13071262)
Supplement: Supplementary file 1 [file genes-13-01262-s001.zip › Supplementary File S2.pdf]

Chromosome X1： CDS sequence of RhoGEF10 gene

ATGTCTACTGTGATGTCTCAGCAACCGCCAAAGGAGGATGAGGAGGAGGAAGATGAGGAG

CAAGGGGAAGAGTTTTTGTTTGAGGACAGCACAGATGAGGAGAAACTTCAGGAGGACAGT

AAAGGGATCGGCTCTGACTCTGTTAAATCTGTCCAGTTGTCAAAAAAGGAGGACAGCAAG

ACATCAGACAGCGTATCACAAACAGGCACAGACAGCACACCACTTCCCAAAACTTCGCCT

CCTGCTGGACAGGAGGGCGTTCCCACCAAGGAAGGAGCTTCAATTTCTGCTGCAGACTGT

GCTAACATTTGGGTACCTTCCAGGGCCTCAGCAGCAGCAGCCGAGCCCCGTACCTCAGCT

CCTTGTGTGGGAGAGGAGCTCCGGGGGCTTCCCGTGGTGCCGTCAGCTGTTGAGACGCTG

GCTGATTCAGGAGACCACAGCAAAGAAATCGTCAAGGAAAGCTCCGGCTCAAGGGACATC

TCTGAAGCTTTACATGTGGCTGAAGCTGAGCCACAGGCGGTGGAGGCTTCAGAGGTGATC

TATGATGATGTTCCAAGTGAGGACCCCCTCTCTCCCGATGAAGATATGATCTATGAAGAT

GTTCAGAGAGACAGTGGTCCTCTGGATGCGGACAACGGGTGGAGCTCCAGTGAGTTTGAA

AGCTACGACGAGCAGTCTGATAACGAGACCAAACTCCCAACACGGAGCAAGCTCTCTCCT

GAAGTGCGTCGGCTGAGGGAACGGTGTGCCAAGACCAAACGTGAACTGGCCATGAGGCTG

TCTGGCAAGCACAACTATGACATCAAGGTTCAACAGCTCATGAAGGCGGCGAGAAGCGGA

ACAAAGGATGGACTTGAAAAGACCAAAATAGCCGTCATGCGAAAAGTTCCCTTCCTGCAG

AGAAAAGAGCAATTGGAGGAGGAAGATGATGCAGGGTACCTGGATGTGGCTGTGTCAGAA

GACAAGCATCCTCCCCCACAGCTGAGCCCCATGCCAGAGGGGCTGACCAGCCACCAGGTT

GTCAGACGCCATATCCTCGGCTCCATCATTCAGAGCGAGCGGAGCTACCTGGAGTCTCTC

AGGAGAATCTTGCAGGAATACCACAGACCATTGATGGAAGCTGATCCACGCATCCTGAGT

CCAAGGAAGATCCGGCCTATATTTTACCGAATCAGGGAGATCACACAGTGCCACTCCATG

TTTCAGATTGCACTGGCGTCACGTGTGGCTGAATGGGACAGCAGTGAGAAAATTGGAGAC

TTATTTGTTGCCTCGTTTTCCAAGTCCATGGTGCTGGATGTATACAGTGATTACGTGAAC

AACTTCACCAATGCCATGGCTCTCATTAAAAAGGCGTGCATGTCCAAACCAGCTTTTCTG

GAATTCTTAAAGAAGAAACAGGCATCTAGTGTTGACCGGATCACTCTTTATGGCCTGATG

GTTAAGCCCATTCAACGATTCCCTCAATTCATTTTGCTTCTGCAGGACATGCTTAAGAAT

ACACCAAAGGGCCACGTGGATCGTCTGCCCCTGCAGCTAGCTCTGACAGAGCTGGAGATG

CTGGCTGAGAAGCTGAATGAACAGAAACGAGTTGCTGACCAGATTGCAGAGACTCAACAA

CTAGCCCGCAGCGTCAGTGATCGCCTGCTCAGTAAGCAACTGAACTCGGAGCAGGGGTCA

CTGGTCCTTTGTGAGACGCTTATTGAGACTGTGTATGGCGAGCGGGGACAAGTCCTGAAG

TCGAAGGAGAGAAAGGTCTTCCTCTTTGATGATGTACTCATCTGTGCCAACATAAATGTC

AAGGGGCCTCCAGATATCAGCAGCCTGGTCCCTGTCGGCCCCAAGTACACCATGAAGTGG

AGCGCCCCCTTACAGCAGGTGCAAGTAGTAGAGGTGGGGCAGGAGGGCTCTCAAAGCAAA

GACACCTTCTTCCCGCAGAGCGGGGCCAAGCGGCCAAGTGGTGCCTGCACTTCAGGTAAA

GCGATCCTTGGCCCTCCACGGCTCTACCAGGAGCTGCAGGAACTGCAGCATGACCTCTCT

GTCGTGGAGGAGGTCACTCTCCTTGTGGGCACGCTGCAGGGGATGTACCAGAACCTGAAC

ACCACTGTGGCCCAGGACTGGTGTCTGGCTCTGCAGAGGCTTATTCGCATCAAAGAAGAA

CAGATCCAGAGTGCTAACAAATGTCGCTTACGCCTGCAGGTGCCTGGCAGGCCAGACAAG

TCAGGGCGTCCTGTCAGTTTCATGGTGGTCCTCAACACTCCCAACCCCCTTAGCAAGATC

TCCTGGGTCAACCGTCTGCACTTGGCCAAGATTGCACTACGAGAGGAGAATTCACCAGGC

TGGTTATGTGGAGAGGACGAAGGGAAGACGATGGCCCCGTTTTGGTGTCCGCTGCTTGCC

TGTCACATGCCTGTCTTTGCTACGAAGTCACCCGAGAGAAAGCTTGAAGCTGCCCTCCAC

AATCCTGTTCACTGTGCATTGTTGGGCTTCTCTGCAGCAAGCACCTCCTTACCTCAGGGC

TACCTCTGGGTGGCTGGCGGAGGAGAAGGTTCTCAGTGGCAGATAGAGATATTTTCCCTC

AACAGGCCCATGCCAAGGGCTGTAAAGAGCCTGCAGGTCGGCTCTGCAGTCCGATGTCTG

GAATATGTGCCAGAACCTTCTCCGACTGAGGAAGCGGAGGCTGGAGTTCACAAGGCCCCC

TCAGAGATCGGTGCCAACATTTGTGTTGGACTAGACGATGGGCGCATACTGGTCTACGGC

AGTGTGGACACTGCAGCACAATGCCTATTGACCCTCCATAACCCAGAGGGCTGCCCTGTG

CTGTGCCTGAAGCACTCCTCCCGCTTCCTGTTTGCAGGCCTACGGAACGGGACCATGATG

GTTTATGGAAGAAGTAACAGTGATGACCTTTGGGACCCTGACTCCTGCAGATGTGTAAGC

CTGGGCTCAGAACCAGTGCGGACACTGTTGGTGTTCGATGACTCAGTGTGGGCAAGCTGC

GGGAACTCGGTCACAGTCATGGACATCTCCAGCCTCACCACTCAGAAGTTTGAAGTTCAT

CCAGACCCGATGGTCAGCGTTGCACACATGGCATGTGCCGGTGGAGGGGTGTGGATGGCA

TTCTCCGAGGGTTCCTCCATTCGTCTCTTTCATACTGAAACACTGGAGCTTCTGCAGGAA

ATCAACATCTCAACACGCTCAACGCTGCTGAACACTGGTCAAAAGAGCATGCGAGTCACG

AGTCTCCTAATTTGCCAGGGGCTGTTATGGGTGGGCACTGCCCAAGGCTTAATTATCACT

CTTCCAGTTCCCAAACTGGAGGGCATCCCCAAAATAACAGGAAAGGGAATGATCTCTCTA

AATGCCCATTGTGGGCCTGTGGACTTCCTGGTAGCCACCACCAGCACTCTGTCTCCTGAA

ATGCTGAAGAGGGATTCTGTCGGGGATGGCCCAGACTCTGCCTGTGGAGGCGAGGACCGC

AGCGACACCTCCTCTCAGGAATCCCTGCAACAGTCCAGCTCCTACCAGGGAGAGGGGAGA

GGCAAAGGACGCGGCGTTCTGCTGCAGTACAAGCTGCGCTCCACGTCAGGACTACCTGGA

CGGCCATTGACGGCACTGGGCGACGAGGCATCGGACTCCTCCCTGGAGTCTTTGGAGCAC

AGCGTGGAAGATGGATCTATCTATGAGCTCAGTGATGACCCAGAAATGTGGGTTCGGGGA

CGTCCTTGTGAGAGAGACGGGGGACGCAGGGACAGGGTGATCTCGGCAGCGGTTATCTCT

GGAGGCAAGGGCTTCCGTAGGCTGAAGGAAGGAGCAGCAGCAGGTACAAGGACAAGTGGG

GAGGGTTCAGAGAATATTCTTATGGTGTGGCAGCTCCCACTGACGGTGTGA

Chromosome Y： CDS sequence of RhoGEF10 gene

GCCACTTCAAATGGTTCTCAATATTCTCATGAACAATTTCCTGAACAATAGG

ATATAATAAACAGTATATGATATGATATATAATACTTTATGTAGCATAATGTATG

TGTTGAATGAATGAATGAACACTGGTACTCTTCTCTTTTATGCCTCCTAAGT

CTTATCATATTGTGCTGTGTGTTCAAGTTTTACGTGTGTATAATGTATAATGC

ATCTCCAGACAGTTCCTGATTAAATAAAGGTTCAACCAAAAATAAAAACTC

TAATGCTGCTAATGCTGATTTTCTCACATGCTGATAACAA
